# Identification and Expression Analysis of bZIP Members under Abiotic Stress in Mung Bean (*Vigna radiata*)

**DOI:** 10.3390/life12070938

**Published:** 2022-06-22

**Authors:** Wenhui Zhang, Shijia Ye, Yanli Du, Qiang Zhao, Jidao Du, Qi Zhang

**Affiliations:** 1 Agronomy College, Heilongjiang Bayi Agricultural University, Daqing 163319, China; hhsilence@byau.edu.cn (W.Z.); ye2608686003@gmail.com (S.Y.); dyl0305@sina.cn (Y.D.); zqiang0416@hotmail.com (Q.Z.); 2 National Coarse Cereals Engineering Research Center, Daqing 163319, China; 3 Research Center of Saline and Alkali Land Improvement Engineering Technology in Heilongjiang Province, Daqing 163319, China

**Keywords:** bZIP genes, bioinformatics, metabolic pathway, mung bean, RNA-seq

## Abstract

The main aim of this study was to identify the bZIP family members in mung bean and explore their expression patterns under several abiotic stresses, with the overarching goal of elucidating their biological functions. Results identified 75 bZIP members in mung bean, which were unevenly distributed in the chromosomes (1–11), and all had a highly conserved bZIP domain. Phylogenetic analysis divided the members into 10 subgroups, with members in the same subgroup having similar structure and motif. The *cis*-acting elements in the promoter region revealed that most of the bZIP members might have the connection with abscisic acid, ethylene, and stress responsive elements. The transcriptome data demonstrated that bZIP members could respond to salt stress at different degrees in leaves, but the expression patterns could vary at different time points under stress. Differentially expressed genes (DEGs), such as *VrbZIP12*, *VrbZIP37*, and *VrZIP45*, were annotated into the plant hormone signal transduction pathway, which might be regulated the expression of abiotic stress-related gene (*ABF*). Quantitative real-time polymerase chain reaction (qRT-PCR) was applied to determine the expression of bZIP members in roots and leaves under drought, alkali, and low-temperature stress. Results showed that bZIP members respond differently to diverse stresses, and their expression was tissue-specific, which suggests that they may have different regulatory mechanism in different tissues. Overall, this study will provide a reference for further research on the functions of bZIP members in mung bean.

## 1. Introduction

Mung bean (*Vigna radiate*), a leguminous crop, has been cultivated in China for more than two thousand years. Given its value in medicine, health care, and nutrition, the demand for mung bean has been rapidly increasing in recent years. However, mung bean production has been maintained at a relatively low level in some major producing areas in north China largely due to the limitation of drought, soil salinization, low temperature in spring, and other adverse environmental conditions. It is well-known that transcription factors (TFs) play important roles in almost all biological processes of plants and are key regulatory factors responding to various signals in plant growth and development as well as environmental stress. Mounting evidence has revealed that transcription factors activate or inhibit transcriptional expression of downstream target genes by binding to promoter or enhancer regions of regulatory genes [1,2,3]. Therefore, this calls for studies to explore the biological characteristics and stress response of transcription factors for breeding and variety improvement of mung bean.

The basic leucine zippers (bZIP) family is one of the largest and most diverse TF families that only exists in eukaryotes [4,5]. All bZIP family members contain a highly conserved bZIP domain consisting of 60–80 amino acids, which is composed of two parts: a highly conserved alkaline region and a variable leucine zipper. The alkaline region, consisting of 16 amino acid residues with a constant N-X7-R/K motif, is responsible for specific recognition and binding to the DNA sequence on the promoter. The leucine zipper, consisting of seven-peptide repeats of leucine or other hydrophobic amino acids, plays an oligomerization function through which the bZIP transcription factor specifically recognizes and forms homologous or heterologous dimers to complete transcriptional activation or inhibition of downstream genes [6,7]. Numerous studies have shown that bZIP transcription factors play an important role in various aspects of plant biological processes, such as embryogenesis [8], seed maturation [9,10], and flower and vascular development [11,12]. On the other hand, bZIP protein is also involved in signal regulation and response to various biotic and abiotic stresses, including high salinity, drought, osmosis, cold stress, and pathogen infection [13]. Studies have shown that the OsbZIP73 gene can improve cold tolerance in rice [14], AtbZIP17 and AtbZIP28 genes can regulate root elongation during stress response [15], and the GmbZIP2 gene characterized from soybean can improve drought and salt tolerance of transgenic tobacco [16].

Over the years, members of the bZIP transcription factor family have been identified or predicted in many eukaryotic genomes [17,18,19,20]. However, no study has identified and characterized the bZIP genes in mung bean. Herein, given the completion of mung bean genome sequencing, we used mung bean genome and transcriptome data to analyze the bZIP family genes from the aspects of systematic evolution, gene structure, protein motif, and chromosome location and then analyzed the expression pattern of the genes under several stresses, including salt, alkali, drought, and low temperature. This study provides a theoretical basis for further exploring the function of bZIP genes in mung bean resistance to various stresses.

## 2. Materials and Methods

### 2.1. Materials and Treatment

Five mung bean seeds (Jilv 10 variety) were sown in a pot (diameter = 8 cm) filled with nutrient soil and cultured in a greenhouse at 22–24 °C and 14 h light and 10 h darkness. After the emergence of mung bean, the two needles were fully expanded, and then 200 mM NaCl, 200 mM mannitol, and 75 mM Na_2_CO_3_ were, respectively, poured onto the root of seedlings. The other seedlings were put in an artificial climate chamber at 4 °C for cold stress, and other conditions were the same as in the greenhouse. Root and leaf samples were collected at 0 h, 6 h, and 24 h time points under 200 mM mannitol, 75 mM Na_2_CO_3_, and 4 °C stress and immediately immersed in liquid nitrogen, followed by storing in the refrigerator at −80 °C for RNA extraction and gene expression analysis. For transcriptome sequencing, leaf samples under 200 mM NaCl stress were collected at 0 h, 6 h, 12 h, and 24 h time points. Notably, all experiments were replicated three times.

### 2.2. Identification and Bioinformatics Analysis of bZIP Family Genes in Mung Bean

We first searched the bZIP genes in mung bean transcriptome data to obtain the gene ID and then searched in NCBI to obtain the gene and protein sequences. Next, the conserved domains of bZIP protein were evaluated using conservative structure domain tools in NCBI database (https://www.ncbi.nlm.nih.gov/Structure/cdd/wrpsb.cgi, accessed on 1 October 2021). Physical and chemical properties of bZIP proteins were analyzed by ExPASy (http://web.expasy.org/protparam/, accessed on 3 October 2021), whereas the motif of bZIP protein was analyzed by MEME (http://meme-suite.org/tools/meme, accessed on 4 October 2021). TBtools was used to visualize the results of protein phylogeny, gene structure, and protein motif analysis [21]. Finally, 2000 bp promoter sequences upstream of the CDS were extracted to predict the *cis*-element using Plant CARE (http://bioinformatics.psb.ugent.be/webtools/plantcare/html/, accessed on 15 October 2021), and then TBtools was applied to visualize the results.

### 2.3. Phylogeny of bZIP Proteins in Mung Bean and Arabidopsis Thaliana

The bZIP protein sequences of *Arabidopsis thaliana* were downloaded from the TAIR database (https://www.arabidopsis.org/, accessed on 12 September 2021). Phylogenetic analysis of bZIP proteins from mung bean and *Arabidopsis thaliana* was carried out by MEGA7.0 adjacency method, with the bootstrap value set to 1000 and other parameters set as default.

### 2.4. Identification and Bioinformatics Analysis of bZIP Family Genes in Mung BeanRNA Extraction and Quantitative Real-Time PCR (qRT-PCR) Analysis

Total RNA was extracted from mung bean tissues using the plant RNA extraction kit (TIANGEN), followed by reverse transcription into cDNA using TaKaRa’s PrimeScriptTM RT Reagent Kit according to the manufacturers’ instructions. Primers for bZIP and reference genes were designed by NCBI primer design tools (https://www.ncbi.nlm.nih.gov/tools/primer-blast, accessed on 10 November 2021) (Table 1). SYBR Green Realtime PCR Master Mix kit was used for quantitative analysis, and each sample was replicated three times. Reaction system 20 μL: 2 μL cDNA, upstream and downstream primers (10 μmol/L) 2 μL, 10 μL SYBR, 6 μL ddH_2_O. Reaction procedure: pre-denaturation at 95 °C for 30 s, denaturation at 95 °C for 10 s, annealing at 60 °C for 30 s, extension at 72 °C for 30 s, cycle 40 times. The genes expression were calculated by 2^-∆∆Ct^ method.

### 2.5. Expression Analysis of bZIP Genes in Mung Bean Leaves under Salt Stress

According to the transcriptome sequencing results, the expression patterns of bZIP family genes in mung bean leaves under salt stress for 0, 6, 12, and 24 h were analyzed using the FPKM value. Heat maps were then generated using R3.4.3 software. The differential gene screening criteria were FDR (false-discovery rate) < 0.01, FC (fold change) > 2.

## 3. Results

### 3.1. Identification and Physicochemical Property Analysis of bZIP Family Members in Mung Bean

**Figure 1:**
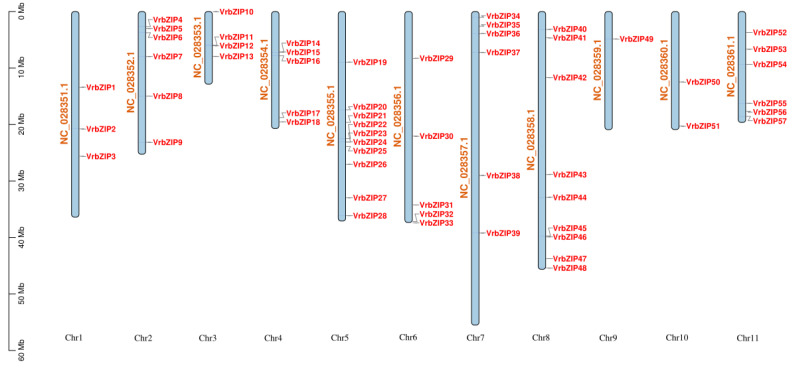
Mapping of bZIP members on mung bean chromosomes.

bZIP gene sequences were first obtained from the NCBI database and then named according to their position on the chromosome, ranging from *VrbZIP1* to *VrbZIP75*. It was evident that the bZIP members were unevenly distributed on the chromosomes (1–11). Specifically, chromosome 5 contained the most bZIP members with ten; followed by chromosome 8 with nine bZIP members; chromosomes 2, 7, and 11 with six bZIP members each; chromosomes 4 and 6 each with five bZIP members; chromosome 3 with four bZIP members; chromosome 1 with three bZIP members; chromosome 10 with two bZIP members; and finally chromosome 9 with one bZIP gene. There were also 18 bZIP members that have not yet been mapped on the chromosomes (Figure 1).

The CDS for distribution of bZIP members ranges from 369 to 2337 bp and encodes 122~778 amino acids. The amino acid length of bZIP members in group D, E, and S is relatively concentrated. The length of amino acids in group S is shorter, distributed between 134 and 200, whereas the amino acid in group D is longer, distributed between 352 and 552. The molecular weight ranges between 13,714.35 and 84,495.52 Da, and the isoelectric point ranges between 2.42 and 10.23. In addition, the basic protein is mainly concentrated in group A, group S, and group H. Results obtained after conducting conserved domain analysis showed that all the 75 mung bean bZIP proteins had bZIP characteristic domains; however, the domain of *VrbZIP37* was partially missing. The basic structure of the conserved domain of mung bean bZIP protein is N-x9-R/K-x7-L-x6-L-x6-L and is composed of basic domain and leucine zipper domain. The basic domain is made up of 20 amino acids, contains conserved structure N-x9-R/K, and is rich in basic amino acids, such as arginine and lysine residues. The N-terminal of the leucine zipper domain is linked to the basic domain, which is composed of three consecutive seven peptides (x6-L-x6-L-x6-L). There is a conserved basic amino acid (leucine) site at the seventh position of each peptide. It is worth noting that most of the conserved domains of mung bean bZIP proteins are 40~59 amino acids; the longest is *VrbZIP32* with 72 amino acids, and *VrbZIP20* is the shortest with only 35 amino acids (Table 2).

### 3.2. Phylogenetic Analysis of bZIP Family Proteins in Mung Bean 

**Figure 2:**
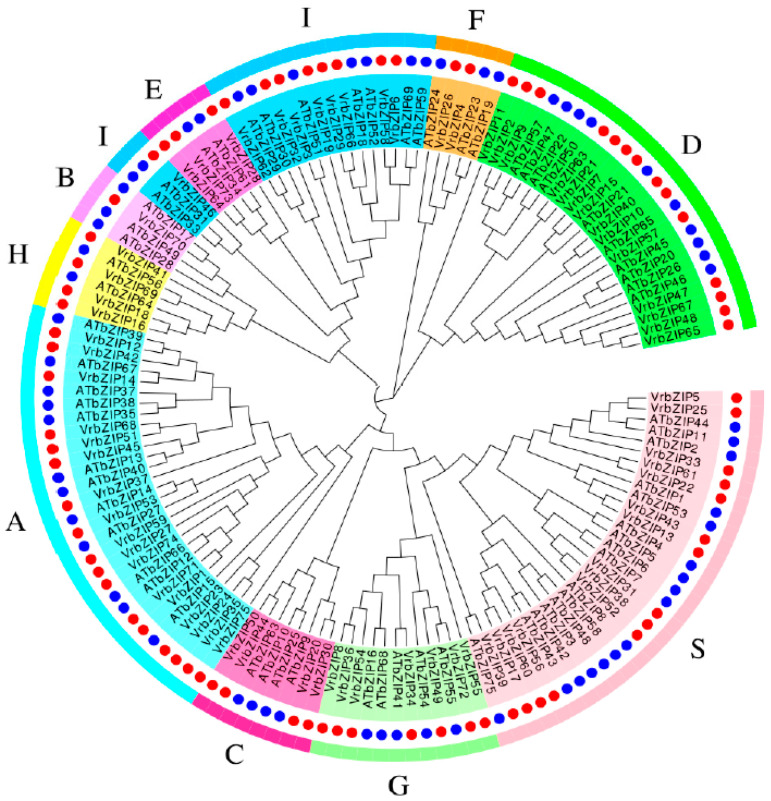
Phylogenetic tree of mung bean and *Arabidopsis* bZIP family proteins.

Multiple sequence alignment and phylogenetic analysis were performed between the bZIP protein sequences of mung bean and *A. thaliana* (Figure 2).

According to the classification results of *A. thaliana*, mung bean bZIP family proteins were divided into 10 groups: A~I and S. Group A had the most members, with a total of 17 accounting for 22.7%; followed by group D and group S, both with 14 bZIP proteins accounting for 18.7%; and group I, with nine members accounting for 12%. Group B had the least members, with only one bZIP protein accounting for 1.3%. The largest group in *A. thaliana* was group S accounting for 22.7%; followed by group A and group I, both accounting for 17.3%; and group D accounting for 13.3%. It was evident that the groups with the largest number of bZIP members were A, D, S, and I, with these four groups containing 72.1% and 70.6% of the total bZIP members in mung bean and *A. Thaliana*, respectively.

### 3.3. Analysis of Conserved Motif and Gene Structure of bZIP Proteins in Mung Bean

**Figure 3:**
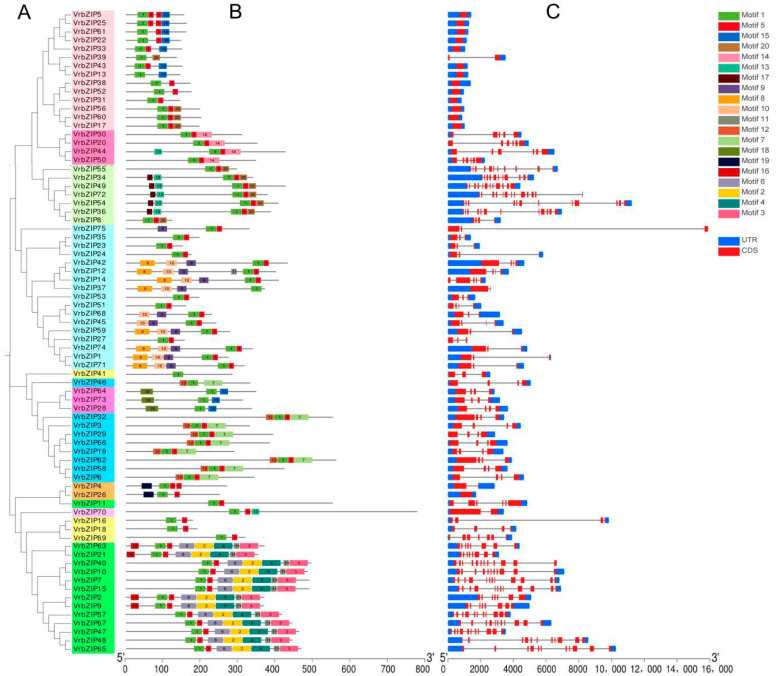
Phylogenetic tree (**A**), conserved motifs (**B**), and gene structure (**C**) among bZIP genes in mung bean.

MEME software was applied to determine the conserved motifs of mung bean bZIP family proteins, with obtained results indicating 20 conserved motifs. The conserved motif, gene structure, and phylogenetic relationship of bZIP proteins were then visually analyzed by TBtools software (Figure 3).

Figure 3A,B show that the motifs contained in the same group of bZIP proteins were similar in type and arrangement order. The members of group S contained only four kinds of motifs, namely motifs 1, 5, 15, and 20. Group C members contained four kinds of motifs, namely motifs 1, 5, 13, and 14, and motif 14 was unique to this group. Group G contained motifs 1, 5, 13, 17, and 20, with motif 13 and motif 17 being specific to this group, where five members contained both of them. In addition, all members of group G contained motif 20, whereas some of the members of group S had motif 20. Group A members had a total of six motifs, namely motifs 1, 5, 8, 9, 10, and 11, with motif 8, 9, and 10 being specific to this group. Group E had three motifs, namely motifs 1, 15, and 18, with motif 15 and motif 18 only existing in this group. With exception of *VrbZIP46*, all other members of group I contained motifs 1, 5, 7, and 12, with motifs 7 and 12 existing in all members of group I and remaining unique to this group. The two members in group F all contained motifs 1, 5, and 19, with motif 19 being unique to this group. Group B, which only had one member (*VrbZIP70*), contained three motifs, namely motifs 1, 5, and 13, with motif 13 being specific to this group. The members of group H only contained motifs 1 and 5 and had no specific motif. Group D had the largest number of motifs, which were motifs 1, 2, 3, 4, 5, 6, 11, and 16. Notably, motifs 2, 3, 4, 6, and 16 were specific to group D. Based on these results, it was evident that motif 1 was common to all members of the mung bean bZIP genes, and motif 5 existed in the vast majority of members. This is because motif 1 is the basic domain of the conserved domain of bZIP proteins, whereas motif 5 is the leucine zipper domain. The two domains are closely associated with each other and determine the binding of bZIP protein to DNA. Moreover, it was found that most of the motifs showed specific distribution in different subfamilies, and most groups had specific motifs different from other groups. Therefore, we speculate that the genes in the same subfamily may have similar functions, whereas the genes in different subfamilies contain specific conservative motif and have a different arrangement order, and thus, they may perform different biological functions.

Based on the analysis of the structure of bZIP family genes in mung bean, it was found that the bZIP genes clustered in the same group had similar gene structure (Figure 3A,C). The number of exons of bZIP family gene in mung bean ranged between one and 18. Group S had the least number of exons, where each gene only had one exon and no intron, with the exception of *VrbZIP39,* which had two exons and one intron. *VrbZIP26,* a member of group F, also had only one exon and no intron. On the other hand, group D and G had the greatest number of exons, with group D exons ranging between 9 and 15 and group G exons ranging between 6 and 18.

### 3.4. Analysis of Promoter Cis-Acting Elements of bZIP Genes in Mung Bean

**Figure 4:**
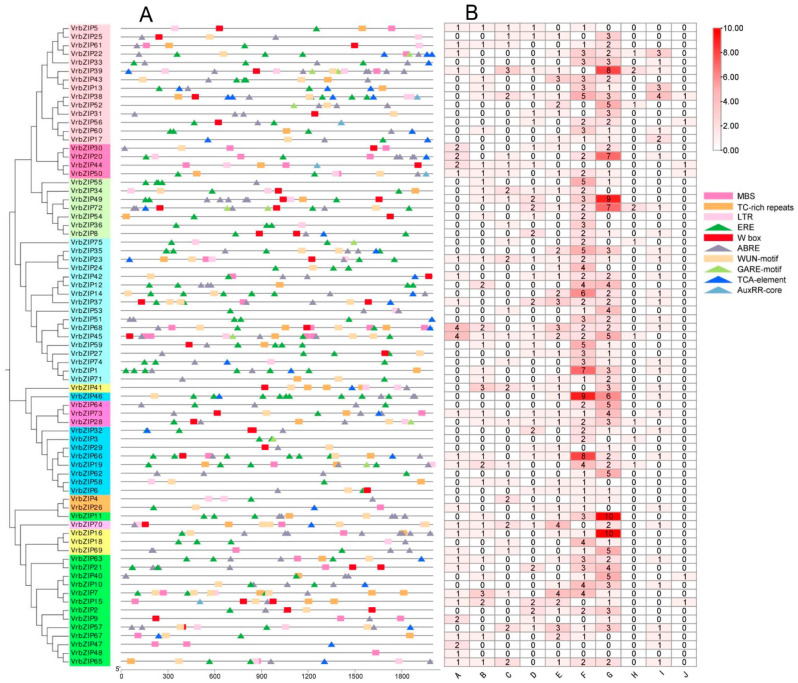
The *cis*-acting elements Distribution of bZIP promoter sequence in mung bean (**A**); Number of *cis*-acting elements (**B**). A, MBS; B, TC-rich repeats; C, LTR; D, Wbox; E, WUN-motif; F, ERE; G, ABRE; H, GARE-motif; I, TCA-element; J, AuxRR-core.

In this study, ten *cis*-acting elements were selected in the 2000 bp upstream CDS of bZIP genes, and a total of 633 elements were obtained (Figure 4). Among them, there were five kinds of hormone response elements, including ERE (ethylene response element), ABRE (abscisic acid response element), GARE-motif (gibberellin response element), TCA-element (salicylic acid response element), and AuxRR-core (auxin response element). There were five *cis*-acting elements associated with defense and stress response: MBS (MYB binding element), TC-rich repeats (defense response element), LTR (low temperature response element), Wbox (trauma and pathogen response element), and WUN-motif (trauma response element) (Figure 4A). In addition, there were 410 hormone response elements accounting for 64.8% of all elements and 223 defense and stress response elements accounting for 35.2% of all elements. Results showed that *VrbZIP38* contained the largest number of elements (18), whereas *VrbZIP48* had the least number of elements (only one component). Among the 75 bZIP genes, 74 genes contained 1 to 16 hormone response elements, among which ethylene response elements and abscisic acid response elements were the most common. Moreover, 70 of the 75 genes contained defense and stress response elements, with the number ranging from 1 to 10 (Figure 4B).

### 3.5. Analysis of Expression Patterns of bZIP Genes in Mung Bean under Salt Stress

**Figure 5:**
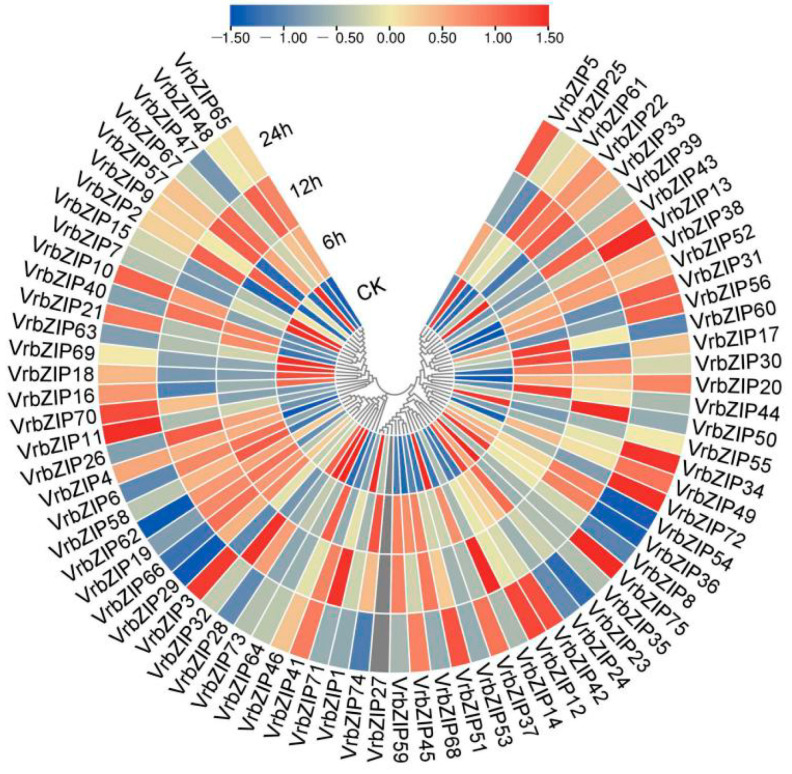
The expression pattern of bZIP genes in mung bean under salt stress. CK:, normal condition; 6 h, NaCl stress for 6 h; 12 h, NaCl stress for 12 h; 24 h, NaCl stress for 24 h.

To analyze the role of bZIP family genes in mung bean response to salt stress, we performed high-throughput RNA sequencing analysis on mung bean leaves under salt stress and then used the “pheatmap” package in R software to generate the expression heat map of bZIP genes (Figure 5). Given that *VrbZIP27* was not expressed under normal conditions and salt stress, we analyzed the expression pattern of the other 74 mung bean genes. Figure 5 shows that bZIP genes of mung bean could respond to salt stress, and there were differences in the expression patterns of different bZIP genes under salt stress. It was found that the expression patterns of bZIP genes in the same group were similar, but there were differences among different groups. Results showed that *VrbZIP1*, *VrbZIP 24*, *VrbZIP 35,* and *VrbZIP 53* genes in group A, which has 17 genes, were downregulated under salt stress. *VrbZIP 23*, *VrbZIP 59*, *VrbZIP 68,* and *VrbZIP 74* had similar expression patterns, where their expression increased at 6 h and 12 h and then decreased at 24 h. There was no significant difference in the expression levels of *VrbZIP 12*, *VrbZIP 42*, *VrbZIP 51,* and *VrbZIP 75* at 6 h and 12 h time points, but the levels increased significantly at 24 h. In addition, *VrbZIP 14* and *VrbZIP 71* as well as *VrbZIP 37* and *VrbZIP45* exhibited the same expression pattern, respectively. The three genes in group E had similar expression patterns. Therefore, considering that the expression patterns of bZIP genes in the same group could be the same, it was evident that bZIP genes in the same group may perform the same biological functions under salt stress.

### 3.6. Screening and Metabolic Pathway Analysis of Differentially Expressed bZIP Genes in Mung Bean under Salt Stress

**Figure 6:**
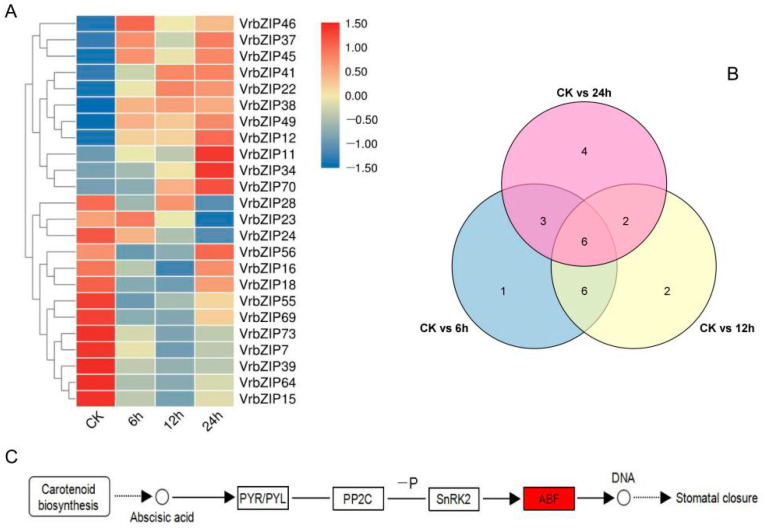
The heat map of differentially expressed bZIP genes under salt stress (**A**), Venn diagram of differentially expressed bZIP genes (**B**), and annotated metabolic pathway of differentially expressed bZIP genes (**C**).

According to the RNA-seq analysis results, genes with fold change ≥ 2 and FDR < 0.01 were regarded as differentially expressed genes. A total of 24 differentially expressed bZIP genes were identified (Figure 6A,B), of which *VrbZIP46*, *VrbZIP37*, *VrbZIP45*, *VrbZIP41*, *VrbZIP22*, *VrbZIP38*, *VrbZIP49*, *VrbZIP12*, *VrbZIP11*, *VrbZIP34,* and *VrbZIP70* were upregulated at different time points under stress. Enrichment analysis showed that *VrbZIP12*, *VrbZIP37,* and *VrbZIP45* genes were enriched in the plant hormone signal transduction pathway mainly in response to the induction of abscisic acid (Figure 6C), which activated the expression of abiotic stress-related gene *ABF* and then regulated the stomatal closure of leaves.

### 3.7. Responses of bZIP Genes to Abiotic Stress

**Figure 7:**
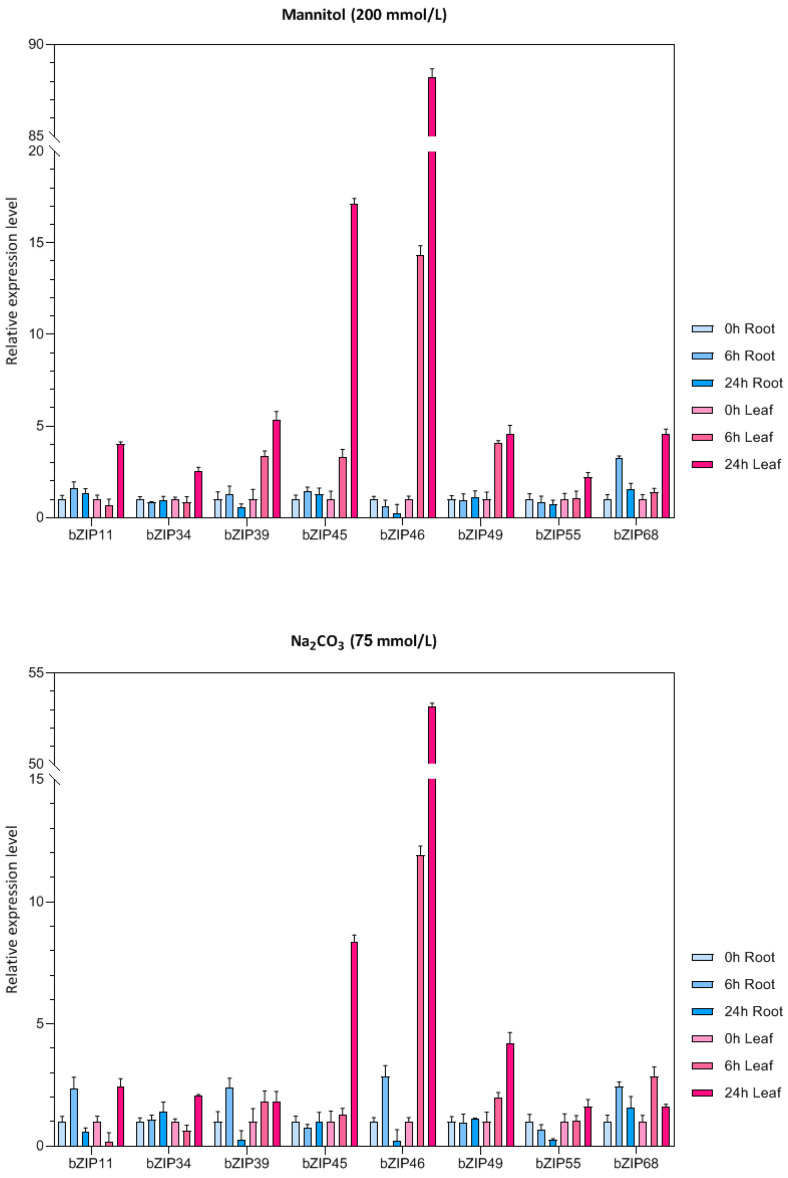

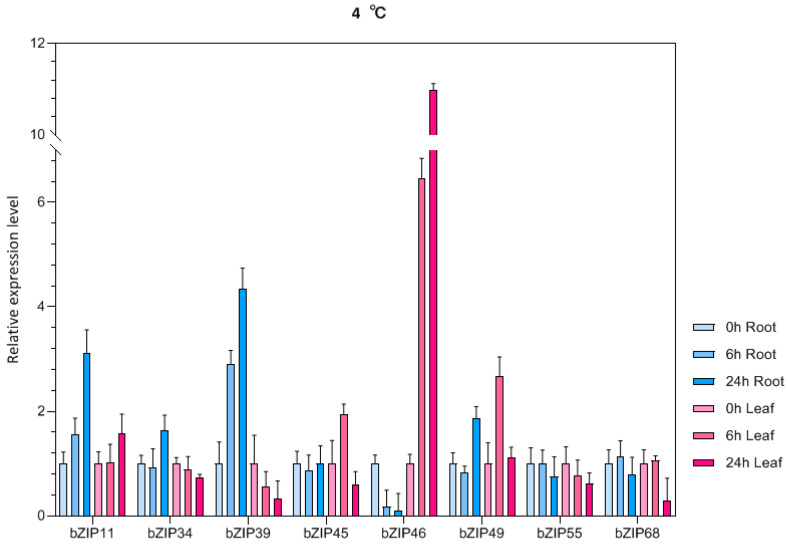
The expression analysis of bZIP genes in mung bean under abiotic stress.

Eight bZIP genes were randomly selected and their expression patterns were analyzed in roots and leaves of mung bean seedlings under drought, low-temperature, alkali, and salt stress (Figure 7). Under drought stress, almost all of the eight bZIP genes were upregulated in different degrees in mung bean leaves compared to the control, and the expression level was the highest after 24 h under stress. However, most genes were not significantly upregulated or downregulated in roots. Under alkali stress, *VrbZIP39*, *VrbZIP45*, *VrbZIP46*, *VrbZIP49,* and *VrbZIP68* were all upregulated in leaves, with the expression of *VrbZIP46* increasing obviously at 6 h and 24 h time points. In the roots, *VrbZIP11*, *VrbZIP39*, *VrbZIP46*, and *VrbZIP68* were first upregulated and then downregulated, whereas there was no significant change was detected in the expressions of *VrbZIP34*, *VrbZIP45*, and *VrbZIP49*. Under low-temperature stress, *VrbZIP46* showed an upregulated expression pattern in leaves, whereas *VrbZIP34*, *VrbZIP39*, *VrbZIP55,* and *VrbZIP68* were all downregulated. In roots, *VrbZIP11* and *VrbZIP39* were upregulated, *VrbZIP46* was obviously downregulated, and there was no obvious change in the expressions of *VrbZIP55* and *VrbZIP68*.

## 4. Discussion

This study identified 75 bZIP genes in mung bean, which is the same number as in *Arabidopsis thaliana* (75) [6] but lower than in soybean (131) [20], rice (89) [17], corn (125) [4], wheat (156) [22], and millet (85) [23]. Notably, the number is greater than that in potatoes (56) [24]. According to systematic evolution analysis of bZIP genes in Arabidopsis and mung bean, it was found that bZIP family genes in mung bean could be divided into 10 groups, similar to Arabidopsis. The intron-exon structure of bZIP genes in mung bean was highly conserved in the same group and had similar intron region distribution. Previous studies reported that the structure of intron-exon is closely associated with the evolution of gene family [25,26,27,28]. In the present study, the number of exons of bZIP genes in mung bean ranged from 1 to 18. Among the 75 genes, 14 genes did not have introns, of which 13 were group S members, and 1 was in group F. Groups D and G had the largest number of exons, with 9–15 exons in group D and 6–18 exons in group G. Similar gene structural diversity has also been found in bZIP family genes in beans and potatoes [20,24], which suggests that different splicing states of exons and introns may be of great significance to the evolution of bZIP genes in mung beans, and there are similarities in the evolution of bZIP genes among different species.

This study also identified and classified 20 conserved motifs of mung bean bZIP proteins. Results showed that all mung bean bZIP proteins contained typical bZIP domain (motif 1), with each group having some common motifs with other groups. Most groups also contained some specific motifs. It is worth mentioning that the bZIP domain, the core of the bZIP family proteins, preferentially binds to the specific *cis*-acting elements of the downstream target gene promoter (such as ABRE). Liu and Chu (2015) demonstrated that different protein motif composition may lead to functional diversity of mung bean bZIP proteins [19]. The gene structure and protein motif distribution of each phylogenetic group were highly conserved, which shows that the genes in the same group are closely associated with the process of evolution.

Furthermore, ten elements were identified in the promoter region of mung bean bZIP genes, and these elements could be divided into two categories: hormone response elements and stress response elements, accounting for 64.8% and 35.2%, respectively. Among the hormone response elements, the number of ERE and ABRE elements was the largest, accounting for 86.1% of the hormone response elements. In addition, 70 of the 75 bZIP genes contained stress response elements. These results suggest that mung bean bZIP genes can participate in the regulation pathway induced by ethylene and abscisic acid, and the existence of a large number of stress response elements lays a foundation for mung bean bZIP genes to participate in various stress responses.

At present, numerous studies have confirmed that bZIP genes play an important role in response to salt and other abiotic stresses in many species [16,29,30,31,32,33]. For multi-gene families, such as bZIP, the analysis of gene expression can provide an important theoretical basis for gene function prediction. The transcriptome sequencing data indicated that mung bean bZIP genes could respond to salt stress in different degrees. Some bZIP genes in the same group had similar expression patterns, such as the three bZIP genes of group E. These results suggest that bZIP genes in the same group may perform the same biological function under salt stress. Studies have shown that PmbZIP genes with the same motifs exhibited similar expression patterns: *PmbZIP31*, *PmbZIP36*, and *PmbZIP41* played more prominent roles in response to freezing stress [34]; 52 putative bZIP genes of *P. Glaucum* were identified, and 9 of them differentially expressed under water stress conditions. These are consistent with our conclusions [35]. It should be noted that some genes in the same group showed varying expression patterns. For example, four bZIP genes in group A were downregulated, and four genes were upregulated at 6 h and 12 h and then downregulated at 24 h. The bZIP gene family is a critical member of the ABA signaling pathway in plants, which plays an important role in plants response to drought [36]. Enrichment analysis of the 24 differentially expressed VrbZIP genes in RNA-seq analysis showed that *VrbZIP12*, *VrbZIP37,* and *VrbZIP45* were annotated in the plant hormone signal transduction pathway, which mainly activates the expression of *ABF*, an abiotic stress-related gene, in response to abscisic acid induction, followed by regulation of stomatal closure in mung bean leaves. Studies have shown that group A bZIP genes in *A.* *thaliana* can activate the expression of abscisic-acid-dependent stress-resistance gene *ABF* under abiotic stress [37]. Accumulating evidence suggests that *ABF* is mainly involved in the response to salt, drought, high-temperature, and low-temperature stresses [38,39], which is consistent with our results. Importantly, two genes (*VrbZIP12* and *VrbZIP37*) that regulate the expression of *ABF* gene belong to group A.

Finally, we analyzed the expression of bZIP genes in roots and leaves of mung bean seedlings under drought, low-temperature, and alkali stress. Results showed that the expression patterns of bZIP genes were different under different stress conditions. For example, *bZIP68* was upregulated under drought and alkali stress but downregulated under low-temperature stress. The expression patterns of the same genes were also different in roots and leaves. Most of the bZIP genes selected in this study were upregulated in leaves under the three kinds of stress but downregulated in roots, indicating that the expression of mung bean bZIP genes under stress is tissue-specific, and the genes may have different regulatory mechanisms in different tissues.

## 5. Conclusions

Seventy-five mung bean bZIP genes were identified, the bZIP genes were unevenly distributed in mung bean chromosomes (1–11), and all had highly conserved bZIP domains. Phylogenetic analysis divided the bZIP genes into 10 groups, with genes in the same group having similar gene structure and protein sequence. Results revealed that the promoter region contained more abscisic acid elements, ethylene response elements, and stress-response elements. Although the mung bean bZIP genes could respond to salt stress in different degrees, the expression patterns of the genes were different at different time points under stress. Moreover, it was evident that the bZIP genes could respond to drought, low-temperature, and alkali stress, and the expression patterns of the genes were tissue-specific. Metabolic pathway enrichment analysis of differentially expressed genes showed that *VrbZIP12*, *VrbZIP37*, and *VrbZIP45* were mainly enriched in the plant hormone signal transduction pathway, which regulates the expression of abiotic stress-related gene *ABF*. Therefore, the three genes can be used as important candidate genes for further gene function verification.

## Figures and Tables

**Table 1:** Primers used for qRT-PCR.

Gene	Forward Primers (5′-3′)	Reverse Primers (5′-3′)
VrbZIP11	CCGACTCTTCACTCTGATCCC	CCTGCTCTACCTCCATGGGT
VrbZIP34	CCGAGTGCTATCCAGCAAAGT	ATGCCATCATTGCCTGAACC
VrbZIP39	GGCAGATTGGAGGATCTCTGG	TGGGAGAGTTGATGGTTTAGTGT
VrbZIP45	AGACCGTCGATGATGTCTGG	CGTGAGGGGCATTGGAATCT
VrbZIP46	ACACTCCGACCCCTCCTATC	GGTCTGCTCGTTGGGTTTCT
VrbZIP49	ACCCTGCAGTTGCTATTGGG	GCCTATTGACATTGCAAGCCC
VrbZIP55	TTCTGCGTCTACTCCGATTCC	GCCAGTACAGCATCCTCAGAT
VrbZIP68	CCTTCTCAAGTCCATCACCCC	GGTAATAGCCTCGTAGCCCTC
Tubulin	GATCTTCCGTCCCGACAACT	AATGGCACACCTGAAACCCT

**Table 2:** Basic information of bZIP family members in mung bean.

Gene Number	Gene ID	Structural Domain (aa)	Chromosomal Location (bp)	CDS	Exon Number	Size (aa)	Molecular Weight (D)	PI
VrbZIP1	XM_022785227.1	203–248	Chr1:13412935..13419247	822	5	273	30,455.24	8.77
VrbZIP2	XM_014666014.2	83–134	Chr1:20775274..20780375	1107	9	368	42,188.04	6.57
VrbZIP3	XM_014647274.2	175–224	Chr1:25617463..25621921	993	4	330	35,793.95	6.78
VrbZIP4	XM_014638991.2	84–150	Chr2:2684648..2687499	810	5	269	29,320.88	6.01
VrbZIP5	XM_014639166.2	32–82	Chr2:3014129..3015546	465	1	154	17,267.41	6.84
VrbZIP6	XM_014663463.2	155–204	Chr2:3749253..3753899	1026	5	341	38,643.78	5.99
VrbZIP7	XM_014636031.2	186–237	Chr2:7950275..7957099	1470	13	489	53,507.07	6.38
VrbZIP8	XM_022775778.1	41–105	Chr2:14961045..14964275	369	6	122	13,714.35	9.13
VrbZIP9	XM_014638786.2	78–128	Chr2:23139450..23144442	1104	12	367	41,473.32	7.73
VrbZIP10	XM_022779041.1	194–243	Chr3:16198..23320	1458	12	485	54,430.47	8.49
VrbZIP11	XM_014640107.2	221–272	Chr3:5971446..5976295	1659	6	552	61,659.10	5.80
VrbZIP12	XM_014640035.2	313–367	Chr3:6059100..6062822	1203	5	400	44,224.20	7.83
VrbZIP13	XM_014639652.2	24–75	Chr3:7927174..7928408	435	1	144	16,286.58	7.87
VrbZIP14	XM_022779735.1	318–365	Chr4:7175101..7177404	1224	5	407	46,183.31	5.13
VrbZIP15	XM_014641492.2	185–235	Chr4:7182698..7189623	1467	15	488	54,141.83	6.58
VrbZIP16	XM_014642005.2	108–158	Ch4:7834772..7844639	534	5	177	19,966.18	9.12
VrbZIP17	XM_014641874.2	85–136	Chr4:18764913..18765929	588	1	195	22,548.07	5.97
VrbZIP18	XM_022779549.1	120–161	Chr4:19550873..19555055	570	4	189	21,465.04	9.83
VrbZIP19	XM_014644694.2	95–144	Chr5:8962527..8967419	867	5	288	32,103.78	5.58
VrbZIP20	XM_014644285.2	197–232	Chr5:17387640..17392584	1053	6	350	38,434.06	6.00
VrbZIP21	XM_014646167.2	67–119	Chr5:19622815..19625932	1059	9	352	39,743.44	7.07
VrbZIP22	XM_014644924.2	32–82	Chr5:22507881..22509023	438	1	145	16,748.77	6.73
VrbZIP23	XM_014645143.2	82–134	Chr5:23096405..23098352	456	3	151	16,753.15	9.94
VrbZIP24	XM_022781035.1	125–170	Chr5:23101214..23107044	525	5	174	19,384.65	9.91
VrbZIP25	XM_014644219.2	31–81	Chr5:23895707..23896990	486	1	161	17,921.00	2.42
VrbZIP26	XM_022781264.1	87–146	Chr5:27052325..27054044	753	1	250	27,593.56	6.45
VrbZIP27	XM_014644051.1	85–130	Chr5:32963429..32964608	468	3	155	18,058.3	9.76
VrbZIP28	XM_014643422.2	185–235	Chr5:36130216..36133900	1008	4	335	37,231.73	7.13
VrbZIP29	XM_014647356.2	195–244	Chr6:8284132..8287012	1179	4	392	42,404.86	6.17
VrbZIP30	XM_014648236.2	147–198	Chr6:22056898..22061393	930	5	309	33,621.12	5.52
VrbZIP31	XM_014648797.2	58–107	Chr6:34234862..34235691	432	1	143	16,954.40	10.00
VrbZIP32	XM_014649544.2	397–469	Chr6:37159446..37162882	1659	4	552	60,573.71	7.68
VrbZIP33	XM_014649426.2	25–75	Chr6:37413761..37414807	447	1	148	17,238.53	9.88
VrbZIP34	XM_014654477.1	256–320	Chr7:1009773..1015431	1017	13	338	35,872.42	5.61
VrbZIP35	XM_022784411.1	126–170	Chr7:2596882..2598280	588	3	195	22,141.67	9.96
VrbZIP36	XM_014654593.2	277–341	Chr7:3911347..3918361	978	13	325	34,708.98	6.18
VrbZIP37	XM_022783046.1	335–359	Chr7:7218005..7221483	1113	7	370	40,329.16	9.75
VrbZIP38	XM_014652907.2	67–118	Chr7:29027489..29028876	516	1	171	19,912.60	9.56
VrbZIP39	XM_022783646.1	28–76	Chr7:39214969..39218506	405	3	134	15,177.45	10.17
VrbZIP40	XM_022785077.1	203–255	Chr8:3177802..3184606	1485	13	494	55,564.89	8.45
VrbZIP41	XM_014656816.2	126–176	Chr8:4647521..4650104	852	3	283	32,145.49	4.99
VrbZIP42	XM_022784953.1	339–393	Chr8:11690917..11695588	1296	5	431	47,609.15	8.76
VrbZIP43	XM_014656646.2	24–74	Chr8:28860359..28861556	450	1	149	16,846.00	5.17
VrbZIP44	XM_014658810.2	224–275	Chr8:32908849..32915369	1278	6	425	45,352.42	5.56
VrbZIP45	XM_014658845.2	160–206	Chr8:39715305..39718710	723	7	240	26,862.45	5.77
VrbZIP46	XM_014657502.2	166–217	Chr8:39904582..39909649	996	4	331	36,551.30	6.22
VrbZIP47	XM_014657576.2	175–222	Chr8:43736639..43740175	1389	11	462	51,367.35	5.87
VrbZIP48	XM_014658700.2	159–211	Chr8:45394513..45403111	1335	13	444	49,199.02	5.76
VrbZIP49	XM_022786075.1	280–343	Chr9:4847038..4851468	1278	13	425	45,488.38	6.13
VrbZIP50	XM_014661380.2	181–218	Chr10:12460328..12462574	1041	6	346	38,098.65	8.24
VrbZIP51	XM_022787071.1	79–125	Chr10:20273437..20275469	480	5	159	18,946.57	9.65
VrbZIP52	XM_014665009.2	76–127	Chr11:3702486..3703453	525	1	174	20,395.12	10.23
VrbZIP53	XM_022775736.1	126–180	Chr11:6665593..6667255	588	3	195	21,724.44	9.61
VrbZIP54	XM_014665463.2	301–365	Chr11:9335409..9346704	1221	14	406	43,311.45	5.90
VrbZIP55	XM_022787574.1	213–263	Chr11:16235928..16242653	888	12	295	33,681.97	5.67
VrbZIP56	XM_014664829.2	85–136	Chr11:17665574..17666561	591	1	196	22,601.39	5.73
VrbZIP57	XM_022775592.1	178–230	Chr11:18570078..18573912	1251	11	416	46,556.78	8.85
VrbZIP58	XM_014666437.2	222–271	Un:200900..204545	1269	5	422	45,733.68	6.02
VrbZIP59	XM_022776281.1	201–243	Un:338441..342976	834	7	277	30,704.20	8.63
VrbZIP60	XM_014666719.2	84–135	Un:100382..101241	603	1	200	23,348.69	6.35
VrbZIP61	XM_014667969.2	31–79	Un:751945..753186	480	1	159	17,967.15	5.25
VrbZIP62	XM_014668844.2	407–458	Un:82484..86411	1686	6	561	61,233.58	6.40
VrbZIP63	XM_022777041.1	78–130	Un:2899..7283	1110	9	369	41,858.61	6.09
VrbZIP64	XM_014668893.2	216–266	Un:732692..736307	1044	5	347	39,271.21	5.48
VrbZIP65	XM_014669108.2	182–234	Un:539901..550192	1404	14	467	51,734.69	5.85
VrbZIP66	XM_014634139.2	185–234	Un:836261..839909	1152	4	383	41,168.21	5.69
VrbZIP67	XM_014634274.2	158–210	Un:36374..42709	1332	13	443	49,224.19	8.85
VrbZIP68	XM_014634567.2	167–207	Un:459429..462604	690	4	229	24,935.03	6.61
VrbZIP69	XM_014634801.2	242–293	Un:339325..343251	954	7	317	34,504.7	5.36
VrbZIP70	XM_014635820.2	277–328	Un:551612..555030	2337	2	778	84,495.52	5.51
VrbZIP71	XM_014635912.2	246–291	Un:1137071..1141720	951	3	316	35,017.23	6.77
VrbZIP72	XM_022777814.1	281–344	Un:765888..774164	1134	18	377	40,660.25	8.56
VrbZIP73	XM_014636225.2	194–244	Un:1420575..1423756	939	4	312	35,868.76	6.03
VrbZIP74	XM_014636721.2	267–312	Un:83487..88340	1014	4	337	37,124.07	6.87
VrbZIP75	XM_014637147.1	214–258	Un:53900..69849	990	3	329	37,431.81	6.86

## Data Availability

All data are contained within the article.
